# Long-term outcomes of destructive seronegative (rheumatoid) arthritis – description of four clinical cases

**DOI:** 10.1186/s12891-016-1067-y

**Published:** 2016-06-03

**Authors:** Elena Nikiphorou, Christopher Sjöwall, Pekka Hannonen, Tuomas Rannio, Tuulikki Sokka

**Affiliations:** Rheumatology/Medicine, Jyväskylä Central Hospital, Keskussairaalantie 19, FI-40620 Jyväskylä, Finland; Rheumatology/AIR, Department of Clinical and Experimental Medicine, Linköping University, SE-581 85 Linköping, Sweden; Faculty of Health Sciences, University of Eastern Finland, Jyväskylä Central Hospital, Keskussairaalantie 19, 40620 Jyväskylä, Finland

**Keywords:** Rheumatoid arthritis, Seronegative, Erosions, Outcomes

## Abstract

**Background:**

Seronegative rheumatoid arthritis is associated with a milder course of progression compared to seropositive disease. However, long-term follow-up data of the clinical course of seronegative rheumatoid arthritis are sparse. Here we describe four cases with a rare disease entity of aggressive destructive seronegative (rheumatoid) arthritis with 20-35 years of follow-up.

**Case presentation:**

The four cases are women with an initial presentation of seronegative rheumatoid arthritis in 1980-1996 and have received disease-modifying anti-rheumatic drugs since the diagnosis. In all cases, the condition has been refractory to treatments and evolved into a severe disease with destructions of the wrists, sub-talar and ankle joints, as well as large joints but not small joints of fingers and toes. All cases are negative with regard to rheumatoid factor, anti-cyclic citrullinated peptide antibodies and antibodies against carbamylated proteins.

**Conclusions:**

This report adds to the existing literature, making the reader aware of this sub-type of inflammatory arthritis which despite being seronegative, can have devastating disease consequences. The report highlights the need for further research into this field in order to better understand this disease sub-type, the pathogenesis, disease course and outcomes.

## Background

Classification criteria for rheumatoid arthritis (RA) have evolved over the decades. Positive serology receives special emphasis in the current criteria [1]. In addition to rheumatoid factor (RF) and anti-cyclic citrullinated peptide (aCCP) antibodies, other RA-related antibodies have recently been identified. These include antibodies against carbamylated proteins (aCarP) [2] and malonaldehyde-acetaldehyde [3], albeit none of these are currently included in classification criteria or used in routine clinical practice.

Autoantibodies are believed to have a pathogenetic role in RA [4]. In studies examining predictors, associating factors, or prevention of RA, seropositive and seronegative groups of patients seem to behave differently [5–7]. In treatment recommendations for RA, seropositivity is recognized as an indicator of severe disease and in these patients, the thresholds for earlier and more intensive/potent disease-modifying treatment are lower [8]. It has become increasingly apparent that our knowledge concerning the pathogenesis, treatment responses and clinical course of seronegative RA, is limited [9, 10].

Up to 20-30 % of patients recruited into RA cohorts and clinical trials are seronegative [11, 12]. An exception is the Finnish Heinola Rheumatism Foundation Hospital early RA cohort from the middle 1970’s, which included long-term (25 years) follow-up of seropositive patients only, as experienced rheumatologists were convinced that seropositive disease (positive RF alone at that time) is the only true presentation of the disease [13]. This formed the basis of our interest in observing the clinical presentation of seronegative RA.

Observations in our early RA cohorts indicate that long-term radiographic outcomes are different between seropositive and seronegative patients [14]. The natural course of seropositive disease is that of progressive erosions [15], while even in the long-term (e.g. over 20 years), seronegative patients do not present with marked erosions [16]. However, among >3000 patients included in the Jyvaskyla Central Hospital clinical RA database since the 1990’s we identified a few consistently RF and aCCP negative individuals with a particular presentation of aggressive, destructive disease. Four cases are presented in detail herein.

## Case presentation

### Case 1

Demographics: 68 year old female, diagnosed at the age of 50 in 10.1996. Shopkeeper, work disabled since the diagnosis; former smoker.

**Fig. 1:**
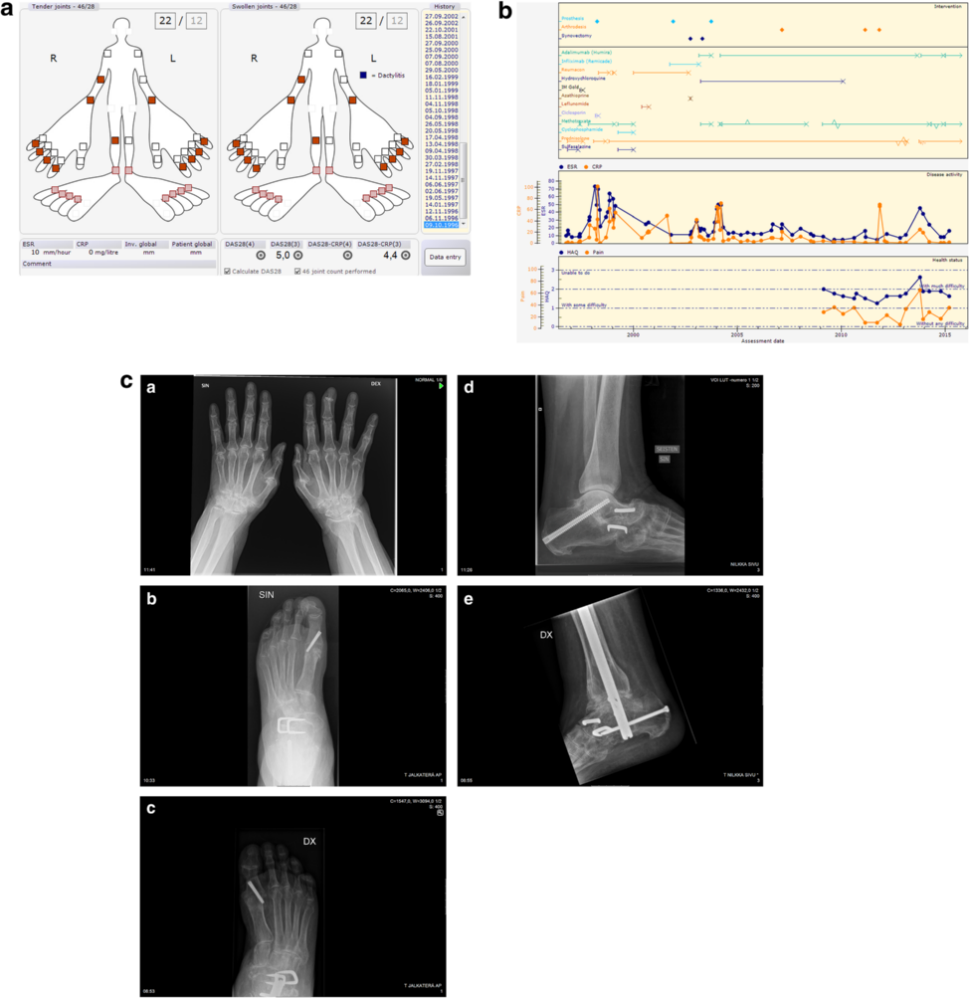
**a** Initial clinical presentation of Patient 1. **b** Progress of Patient 1. **c** 1-5. Most recent radiographs of Patient 1 (16-19 years from diagnosis)

Initial presentation: Swelling and tenderness starting in the right knee in 01.1996. Intra-articular glucocorticoid injections administered into the right knee four times, until arthroscopy in 08.1996 with macro- and microscopic finding of synovitis. Joint symptoms gradually evolved resulting in the diagnosis of clinical polyarthritis in 10.1996. See Fig. 1a.

Comorbidities and Joint surgery: See Table 1.

**Table 1:** Medical history of Patient 1

Data extracted	25.08.2015
ID	Case 1
Age, Gender	68, Female
Work status	Pensioner since diagnosis
Diagnosis	Rheumatoid Arthritis
Diagnosis criteria	- Symptoms: 1.1996
	- Clinical diagnosis of RA: 10.1996
Highest RF (IgM)	Negative (9) 3.2012
Highest (aCCP)	Negative (4) 8.2009
aCarP	Negative 7.2015
HLA-B27	Positive
Drug (now)	Methotrexate 1.2015
	- 7,50 mg Subcutaneous Once a week
	Adalimumab (Humira) 1.2015
	- 40,00 mg Subcutaneous Every 2. week
	Prednisolone 10.2013
	- 5,00 mg Peroral Every day
Comorbidity	Vertebral fracture 10.2005
	Herpes zoster infection 9.2002
	Arm fracture, not wrist 8.2001
	Osteopenia by DXA 2001
	Cholelithiasis 3.1998
	Arterial hypertension 11.1996
	Fibromyalgia 7.1990
	Lumbago/dorsalgia/sciatica 1980
	Migraine - Before rheuma diagnosis
Surgery	Left ankle Arthrodesis 11.2011
	Right ankle Arthrodesis 3.2011
	Left MTP Arthrodesis 3.2007
	Right MTP Arthrodesis 3.2007
	Left knee Arthroplasty 10.2003
	Left knee Synovectomy 5.2003
	Right knee Arthroscopy and Synovectomy 10.2002
	Right knee Rearthroplasty 12.2001
	Right knee Arthroplasty 4.1998
	Right knee Other surgery 8.1996

Medications: Current medications, see Table 1. Previous synthetic and biologic disease modifying anti rheumatic drug (DMARD) treatments were (in order of use): sulfasalazine, gold intra muscular (IM), podofyllotoxin (Reumacon), ciclosporin, cyclosphosphamide, leflunomide, infliximab, azathioprine, and hydroxychloroquine, see Fig. 1b.

Progress: See Fig. 1b. Most recent values for patient reported outcomes and disease activity: HAQ 1.63, pain 35, fatigue 6, patient global 30, ESR 16, CRP 2, DAS28 3.4 indicating moderate disease activity.

Radiographic features: Radiographs on presentation showed no joint damage. The most recent radiographs of hands, wrists, feet, and ankles, taken 16-19 years after diagnosis, are shown in Fig. 1c. Prominent features include joint space narrowing and subluxation of bones in left wrist and meta carpo phalangeal MCP joints, destruction and operation of subtalar, talonavicular and naviculo-cuneiforme joints, as well as right ankle. Typical erosions, seen as in seropositive RA patients are missing.

### Case 2

Demographics: 39 year old female, diagnosed at the age of 16 in 07.1992. Working as a civil engineer. Never smoked.

**Fig. 2:**
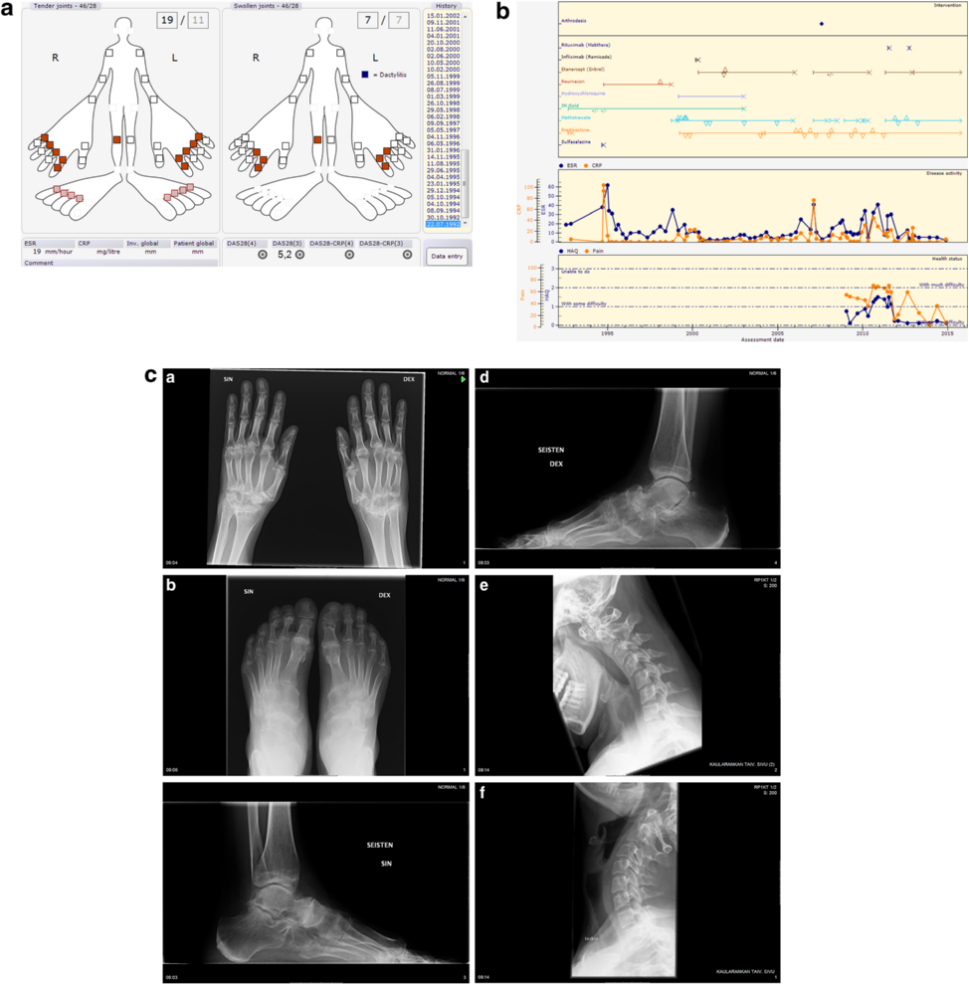
**a** Initial clinical presentation of Patient 2. **b** Progress of Patient 2. **c** 1-6. Most recent radiographs of Patient 2 (20-23 years from diagnosis; neck radiographs in 2007)

Initial presentation: The patient sustained a left knee strain while running in 3.1992; symptoms spontaneously resolved within 3 weeks. She was noted to have hyperextension of the left knee in 6.1992 with 7.1992 with synovial fluid aspiration showing 14.000 leucocytes (98 % granulocytes) and a clinical presentation of polyarthritis as shown in Fig. 2a.

Comorbidities and Joint surgery: See Table 2.

**Table 2:** Medical history of Patient 2

Data extracted	15.09.2015
ID	Case 2
Age, Gender	39, Female
Work status	Full-time work
Diagnosis	Rheumatoid Arthritis
Diagnosis criteria	- Symptoms: 3.1992
	- Clinical diagnosis of RA: 7.1992
Highest RF (IgM)	Negative (8) 2.2012
Highest (aCCP)	Negative (0) 2.2012
aCarP	Negative 7.2015
HLA-B27	Negative
Drug (now)	Etanercept (Enbrel) 1.2013
	- 50,00 mg Subcutaneous Once a week
	Methotrexate 5.2011
	- 15,00 mg Subcutaneous Once a week
	Prednisolone 4.2004
	- 5,00 mg Peroral Every day
Comorbidity	Neutropenia 12.2012 - 12.2012
	Viral infection 12.2012
	Osteopenia by bone density by DXA 3.2012
Surgery	Neck Arthrodesis 8.2007
	Right wrist Tenosynovectomy 10.1995

Medications. Current medications see Table 2. Previous DMARD treatments included (in order of use): i.m. gold, sulphasalazine, podofyllotoxin (Reumacon), hydroxychloroquine, infliximab, and rituximab see Fig. 2b.

Progress: See Fig. 2b. Most recent values for patient reported outcomes and disease activity: HAQ 0.13, pain 8, fatigue 12, patient global 9, ESR 2, CRP 5, DAS28 2.1 indicating remission.

Radiographic features: Initial joint radiographs on presentation showed no damage. The most recent radiographs 20-23 years after diagnosis are presented in Fig. 2c. The prominent features are damage in wrists, subluxation of right MCP II, damage of talonavicular and naviculo-cuneiform joints, but minimal erosive changes characteristic of seropositive RA.

Radiographs of the neck in 2007 indicated a sliding atlanto axial subluxation of 7 mm in head bending forward –position. An MRI of the neck showed no active pannus and no erosions in the dens.

### Case 3

Demographics: 63 year old female, diagnosed at the age of 28 in 05.1980. Cashier, work disabled since September 1989. Never smoked.

**Fig. 3:**
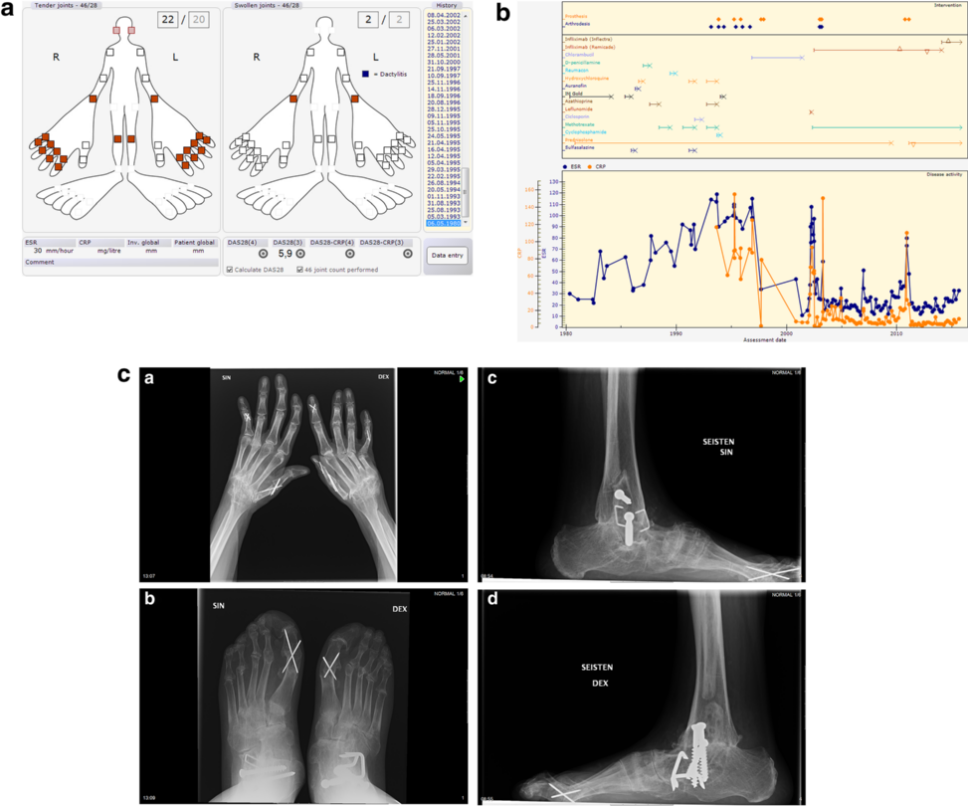
**a** Initial clinical presentation of Patient 3. **b** Progress of Patient 3. **c **1-4. Most recent radiographs of Patient 3 (32-35 years from diagnosis)

Initial presentation: Temporary pains and aches in knees, wrists, and fingers since the age of 15, and increasing several months prior to the diagnosis in 05.1980 when she presented with a polyarthritis as shown in Fig. 3a.

Comorbidities and Joint surgery: See Table 3.

**Table 3:** Medical history of Patient 3

Data extracted	14.09.2015
ID	Case 3
Age, Gender	63, Female
Work status	Disabled pensioner since 9.1989
Diagnosis	Rheumatoid Arthritis
Diagnosis criteria	- Symptoms: 6.1979
	- Clinical diagnosis of RA: 5.1980
Highest RF (IgM)	Negative (9) 6.2014
Highest (aCCP)	Negative (0) 9.2014
aCarP	Negative 7.2015
HLA-B27	Negative
Drug (now)	Infliximab (Inflectra) 2.2014
	- 300,00 mg Intravenous Every 8. week
	Prednisolone 2.2011
	- 2,50 mg Peroral Every day
	Methotrexate 5.2002
	- 10,00 mg Peroral Once a week
Comorbidity	Hyperlipidemia 4.2011
	Hypothyroidism 4.2011
	Osteoporosis by DXA 11.1997
	Arterial hypertension 6.2011
Surgery	Left elbow Prosthesis 2.2011
	Right elbow Prosthesis 04.10.2010
	Left MCP-1 Arthrodesis 3.2003
	Left MCP-4 Prosthesis 3.2003
	Left PIP-1 Prosthesis 3.2003
	Left PIP-5 Arthrodesis 3.2003
	Right DIP-2 Arthrodesis 1.2003
	Right MCP-1 Arthrodesis 1.2003
	Right MCP-3 Prosthesis 1.2003
	Right MCP-4 Prosthesis 1.2003
	Right PIP-5 Arthrodesis 1.2003
	Right shoulder Prosthesis 12.1997
	Left shoulder Prosthesis 9.1997
	Right ankle Arthrodesis 9.1996
	Right ankle Arthrodesis 1.1996
	Left hip Prosthesis 11.1995
	Right ankle Arthrodesis 6.1995
	Right hip Prosthesis 4.1995
	Left ankle Arthrodesis 5.1994
	Right MCP-3 Prosthesis 11.1993
	Right MCP-4 Prosthesis 11.1993
	Right MCP-5 Prosthesis 11.1993
	Right PIP-1 Arthrodesis 11.1993
	Left ankle Arthrodesis 3.1993
	Right MTP – Joint resection 1990
	Left MTP – Joint resection 1990
	Right wrist Tenosynovectomy 2.1981

Medications. Current medications are presented in Table 3. Previous DMARD treatments were (in order): gold i.m., sulphasalazine, auranofin, hydroxychloroquine, D-penicillamine, azathioprine, podofyllotoxin (Reumacon), ciclosporin, cyclophosphamide, chlorambucil, leflunomide, and infliximab (Remicade) see Fig. 3b.

Progress: See Fig. 3b. Most recent values for patient reported outcomes and disease activity: HAQ 1.38, pain 0, fatigue 0, patient global 0, ESR 33, CRP 10, DAS28 3.0 indicating low disease activity.

Radiographic features: No radiographic joint damage was seen at initial presentation. The most recent radiographs 32-35 years after diagnosis are presented in Fig. 3c and show prominent damage in the, wrists, ankle/subtalar/upper forefeet areas, as well as status post orthopaedic surgery in many joints.

### Case 4

Demographics: 60 year old female. Diagnosed at the age of 28 in 12.1982. Waitress, work disabled since 1995, former smoker.

**Fig. 4:**
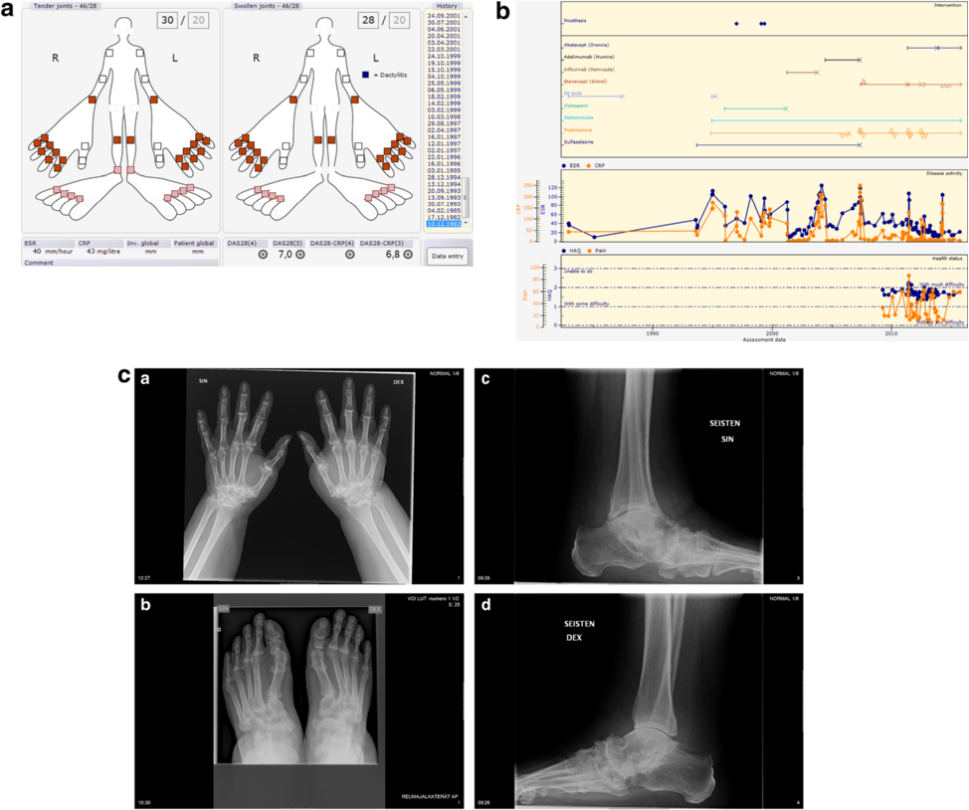
**a** Initial presentation of Patient 4. **b** Progress of Patient 4. **c** 1-4. Most recent radiographs of Patient 4 (30-33 years from diagnosis)

Initial presentation: Symptoms started two years before diagnosis, with synovitis in both knees. Two months prior to diagnosis, symptoms began in other joints with clinical polyarthritis in 12.1982 as presented in Fig. 4a.

Comorbidities and Joint surgery: See Table 4.

**Table 4:** Medical history of Patient 4

Data extracted	14.09.2015
ID	Case 4
Age, Gender	61, Female
Work status	Pensioner since 1995
Diagnosis	Rheumatoid Arthritis
Diagnosis criteria	- Symptoms: 6.1980
	- Clinical diagnosis of RA: 12.1982
Highest RF (IgM)	Negative (8) 4.2009
Highest (aCCP)	Negative (4) 4.2009
aCarP	Negative 7.2015
HLA-B27	Positive
Drug (now)	Abatacept (Orencia) 12.2013
	- 125,00 mg Subcutaneous Every week
	Etanercept (Enbrel) 10.2012
	- 50,00 mg Subcutaneous Every 10. day
	Prednisolone 5.2007
	- 5,00 mg Peroral Every day
	Methotrexate 12.1994
	- 20,00 mg Peroral Once a week
Comorbidity	Colon diverticulitis 8.2013
	Urinary tract infection 7.2012
	Bacterial infection 6.2012
	Pulmonary embolism 8.2011
	Pulmonary embolism 12.2010
	Asthma 11.2008
	Hyperlipidemia 1.2008
	Arterial hypertension 6.2007
	Osteoporosis by DXA measurement 1.1998
Surgery	Right hip Prosthesis 5.1999
	Left knee Prosthesis 2.1999
	Left hip Prosthesis 1.1997

Treatment: Current medications are presented in Table 4. Previous DMARD treatments were (in order): Im gold, sulfasalazine, cyclosporine, infliximab, and adalimumab, see Fig. 4b.

Progress: See Fig. 4b. Most recent values for patient reported outcomes and disease activity: HAQ 1.75, pain 59, fatigue 40, patient global 50, ESR 22, CRP 5, DAS28 2.9 indication low disease activity.

Radiographic features: The most recent radiographs of hands, wrists, feet, and ankles, 30-33 years after diagnosis, are presented in Fig. 4c. Destruction of wrists and ankle/subtalar area are prominent, with minimal/no erosions typical for seropositive RA.

## Discussion

These cases all share a common feature: that of a severe, destructive disease in seronegative RA with involvement primarily of the wrists, sub-talar and ankle joints, as well as large joints. All these patients were negative with regard to RF, aCCP, and aCarP. Two cases were HLA-B27 positive but despite this, the clinical presentation (signs and symptoms) and radiographs of the sacro-iliac joints did not support a diagnosis of ankylosing spondylitis (AS) or other spondyloarthritides in these individuals.

Seronegative RA has been in focus of only a few cohorts and rarely in detail, to reveal various aspects of outcomes [9]. Again, an exception is from the Heinola group, which reported long-term outcomes of non-specific seronegative oligoarthritis in patients with a 23 year follow-up [16]. Based on patient history, radiographs and clinical status at the follow-up visit, they re-classified the 64 patients and found one case each of RA, systemic lupus erythematosus and ankylosing spondylitis, two cases of post-traumatic arthritis, four cases of osteoarthritis, and six cases of possible reactive arthritis. Of the remaining 49 patients, 15 were HLA-B27 positive and 16 had at least one of the psoriasis-related HLA antigens. Seven patients had minor erosions in their hands or feet joints. One HLA-B27 positive patient had developed bilateral sacroilitis by the evaluation at 23 years. Functional capacity of the patients was well retained. Compared to this Heinola seronegative cohort, our patients present a more severe destructive disease, and to date, remain un-classified.

To our experience, patients with destructive seronegative (RF, aCCP negative) RA - as this disease entity can be referred to - are rare. Among our approximately 3000 RA patients, of whom 30 % are seronegative, only a few seem to present with such degree of destructive disease. However, over the years it is possible that similar cases have been missed, as prior to the era of aCCP analyses, a proportion of RF negative cases demonstrated a typical RF positive course and were later revealed to be aCCP positive. Although destructive seronegative RA is rare, it can have devastating consequences and early recognition and intensive treatment is paramount.

The current clinical status of the patients presented in this report indicates considerable functional loss with a HAQ > 1.5 in three of the four patients. Despite this, disease activity appears to be under at least some degree of control with DAS28 values between 2.1 to 3.4. Current treatment in all four patients includes prednisolone, methotrexate and biologic agents (combination of two biologic agents in Case 4).

The radiological progression of joint damage in these patients presents major cartilage destruction and loss and with minor bony erosions of joints. In fact, it could even be argued that the radiographic destruction seen in these cases resembles that of advanced seronegative juvenile polyarthritis.

## Conclusions

Through a detailed description of four destructive seronegative (for RF, aCCP and aCarP) RA cases from disease-onset and up to 35 years from diagnosis, this report could shed more light on the disease presentation, course and outcomes of such patients. We hope to inform the reader of this particular sub-type of inflammatory arthritis which can result in devastating patient outcomes and therefore needs prompt identification and treatment, similar to seropositive disease. This report justifies the undertaking of further research on the pathogenesis and identification of possible biomarkers for this type of arthropathy, which could be invaluable in firstly understanding disease behavior and course and secondly in treatment stratification.

## Abbreviations

aCarP, anti-carbamylated protein antibodies; aCCP, anti-cyclic citrullinated peptide antibodies; CRP, C-reactive protein; DAS, disease activity score; DMARD, disease modifying anti rheumatic drug; ESR, erythrocyte sedimentation rate; HLA, human leucocyte antigen; IM/i.m, intra muscular; MCP, meta carpo phalangeal; RA, rheumatoid arthritis; RF, rheumatoid factor.
